# A data driven learning approach for the assessment of data quality

**DOI:** 10.1186/s12911-021-01656-x

**Published:** 2021-11-01

**Authors:** Erik Tute, Nagarajan Ganapathy, Antje Wulff

**Affiliations:** Peter L. Reichertz Institute for Medical Informatics of TU Braunschweig and Hannover Medical School, Carl-Neuberg-Str. 1, 30625 Hannover, Germany

**Keywords:** Information science, Data quality, Data aggregation, Knowledge bases, Machine learning

## Abstract

**Background:**

Data quality assessment is important but complex and task dependent. Identifying suitable measurement methods and reference ranges for assessing their results is challenging. Manually inspecting the measurement results and current data driven approaches for learning which results indicate data quality issues have considerable limitations, e.g. to identify task dependent thresholds for measurement results that indicate data quality issues.

**Objectives:**

To explore the applicability and potential benefits of a data driven approach to learn task dependent knowledge about suitable measurement methods and assessment of their results. Such knowledge could be useful for others to determine whether a local data stock is suitable for a given task.

**Methods:**

We started by creating artificial data with previously defined data quality issues and applied a set of generic measurement methods on this data (e.g. a method to count the number of values in a certain variable or the mean value of the values). We trained decision trees on exported measurement methods’ results and corresponding outcome data (data that indicated the data’s suitability for a use case). For evaluation, we derived rules for potential measurement methods and reference values from the decision trees and compared these regarding their coverage of the true data quality issues artificially created in the dataset. Three researchers independently derived these rules. One with knowledge about present data quality issues and two without.

**Results:**

Our self-trained decision trees were able to indicate rules for 12 of 19 previously defined data quality issues. Learned knowledge about measurement methods and their assessment was complementary to manual interpretation of measurement methods’ results.

**Conclusions:**

Our data driven approach derives sensible knowledge for task dependent data quality assessment and complements other current approaches. Based on labeled measurement methods’ results as training data, our approach successfully suggested applicable rules for checking data quality characteristics that determine whether a dataset is suitable for a given task.

**Supplementary Information:**

The online version contains supplementary material available at 10.1186/s12911-021-01656-x.

## Background

Reuse of electronic patient data, e.g. for medical research, is an active field of research [1, 2]. One challenge for valuable data reuse is data quality (DQ), where DQ denotes the ability of data to “serve the needs of a given user pursuing specific goals” [3]. Thus, DQ-assessment (DQA) is dependent on the goals the data user is pursuing (also known as “task dependency”). Established DQA reporting standards determining relevant measurement methods (MM) for different tasks are missing [3–7]. A MM is a specification of a method that quantifies a characteristic of a dataset (cf. [8]). An exemplary MM could be a method counting the number of existing values for a variable. The selection of relevant MMs for DQA in a specific task alone is challenging. But complexity is even increased because different reference ranges indicating DQ-issues can be suitable. Even for a simple example, such as the patient height. The range for valid values, e.g. 250 cm could define the threshold for an implausible magnitude. The number of implausible values in a dataset that is tolerable could be five implausible values per 1000 values (another possible type of threshold). Considering soft and hard limits (cf. [9]) even more reference ranges seem sensible, e.g. a magnitude of more than 230 cm is a suspicious (soft-limit) and more than 300 cm is a wrong value (hard-limit) for which different numbers for violations are tolerable. Even with a limited amount of variables, the number of MMs and possible assessments of their results grow quickly. Which MMs to apply in which situations and how to assess their results is what we refer to as “DQA-knowledge”. So far, applied DQA-knowledge is often intangible and based on experts’ personal experience [6, 8, 10]. Our previous work proposed an interoperable knowledge-based approach to DQA to support the application and collaborative governance of formalized task and domain dependent DQA-knowledge [11]. The work we report on here addresses the challenge of learning such DQA-knowledge out of data. A common approach to identify relevant MMs and reference ranges for a given purpose is to review literature on DQA in similar situations, to study published DQA frameworks and to interview experts (cf. [12–16]). Complementing this with data driven methods, which are less dependent on experts’ opinions and that better support collaborative learning of DQA-knowledge is desirable. Johnson et al. proposed a method to quantify the impact of DQ in different variables on a given purpose based on a linear regression fitted with MM-results and outcome data [17]. Their method allows to quantify the task dependent impact for MMs with results suitable for linear regressions but does not address thresholds. Other authors employ data driven methods for DQA directly on the data (in contrast to applying it on MM-results) to identify deviant records [18, 19], deviant distributions [20] or to identify clusters representing DQ-issues [21] independent of the relevance for the task. In our work, we examine a new approach to derive DQA-knowledge from shared MM-results and corresponding outcome data.

### Objectives

In this work, we aim at assessing the applicability of a data driven approach to support the learning of tangible DQA-knowledge, i.e. reference ranges of MM-results and their prioritization depending on result's values. In detail, we address the following questions:

1. Are machine learning methods able to derive sensible DQA-knowledge from exported MM-results and corresponding outcome data?
2. Does applying machine learning complement the manual identification of DQA-knowledge?

## Methods

**Fig. 1:**
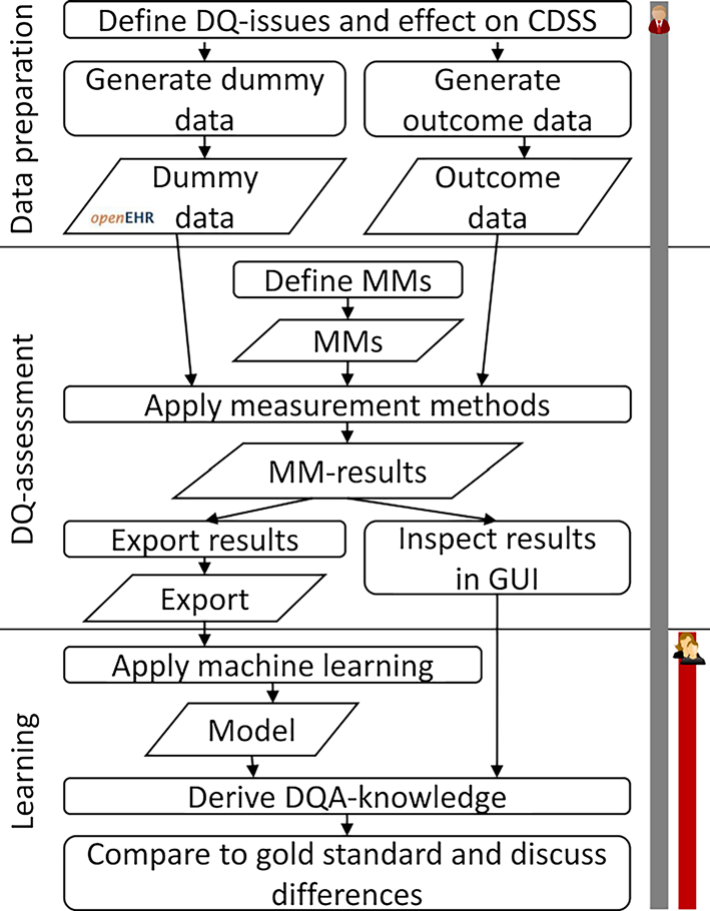
Process overview—vertical lines on the right side indicate the participation of researchers in the different phases of the work. Grey = ET, red = NG and AW

We imagine a fictive scenario of a clinical decision support system (CDSS) in cardiology predicting a score for a patient based on anamnesis data. We assume that the DQ of its input data influences the quality of its predictions (cf. [17, 22]). Our fictive CDSS is a black box, so that we don’t know its underlying algorithms. In a real world scenario, we would not know by which mechanisms DQ affects correct or incorrect predictions. In our fictive scenario, we predefine how DQ affects CDSS success. This enables us to assess whether the DQA-knowledge derived by our data driven approach is correct. Figure 1 depicts our overall proceeding. One of the authors (ET) performed the data preparation and DQA steps. Three researchers independently derived DQA-knowledge from the machine learning results. Thus, one did this knowing the present data quality issues and two without having any prior knowledge about them.

### Data preparation

#### Generating clinical dummy data

First step was the generation of 10,500 openEHR compositions (comparable to a message or document) of dummy data using an open source dummy data generator [23] (The script used to generate the dummy data for this work is available in commit f28dedd0 from 2021-02-19). Each composition represented one clinical case/patient with multiple variables and values. The cases were distributed to 20 fictive hospital sites with a different number of cases for each hospital. Generated compositions complied with the openEHR specifications [24] and based on a real world specification for a cardiology anamnesis [25]. The data was stored in a local installation of an openEHR data repository [26]. The generated data contained different kinds of DQ-issues. These issues were defined in advance based on knowledge from literature and were intended to cover a range from obvious issue to very hard to detect. An example for an obvious DQ-issue is a case without any blood pressure measurements, which causes the fictive CDSS to fail. A possibly hard-to-detect issue example would be a case with less than ten heart rate measurements, which only causes a slightly lower CDSS performance. Additional file 1: Appendix A lists the number of occurrences, the issue’s effect and an explanation for each defined DQ-issue.

#### Generating outcome data

Key to our learning approach is to have a dataset with labeled data, i.e. we need a variable indicating whether the task, e.g. a CDSS, succeeds or not in granular subsets of the data. In a real world scenario, information on correctness of the CDSS prediction would base on data retrieved from the patients’ EHRs. For example, we could retrieve the predictions and the actual outcomes from the data repository and apply a function to decide whether the prediction was good enough or not. Since our CDSS is fictive, we generated our outcome and added it to the data sets as follows. For each case, a script checked the data regarding the initially defined DQ-issues (cf. Fig. 1) to determine the probability of a correct prediction. For example, an issue could be that a patient’s data is missing a needed information. The fictive CDSS could use information about patient gender and predictions could be less accurate if this information was missing for a patient (Additional file 1: Appendix A lists all issues). The likelihood for a correct prediction in a patient with perfect data quality was defined as 95%. For each issue present in a patient's data, the script reduced the success probability by a factor specific to that issue (and counted that this issue influenced a patient’s outcome). Thus, bad DQ in a patient’s data led to a lower probability of a correct CDSS prediction for this patient. For our gender issue, this factor is set to 0.95. A patient with missing data about gender and apart from that perfect data would have a probability for a correct CDSS prediction of 0.9025 (factor 0.95 applied to reduce the initial 95% chance for correct prediction). Based on this probability we generated the outcome value for each patient as *true* for a correct CDSS prediction or *false*. In a real world scenario, each patient would have one outcome value indicating whether the CDSS prediction was correct or not. We generated three outcome values for each patient. Now a patient with probability for a correct CDSS prediction of 0.66 could have the outcome values: *Outcome 1* = true, *Outcome 2* = false, *Outcome 3* = true. We did this to perform the subsequent steps three times with different outcome data in order to reduce the chance that good results in our work occur just due to a lucky coincidence in outcome generation (we refer to the different sets of outcome data as *Outcome 1* to *Outcome 3*). The script for generating the outcome values is available in Additional file 2: Appendix B. We used the counts about DQ-issue occurrences influencing outcome generation to validate the correct implementation of outcome generation by comparing the counted DQ-issues that had an effect on outcome to expected counts (expected based on DQ-issue frequency in dummy data creation—cf. Additional file 1: Appendix A).

### DQ-assessment

#### Defining and applying measurement methods

We used the open source tool openCQA ([11, 27] commit c0a8a784 from 2021-02-19) for DQA. We generated simple MMs based on variables’ datatypes calculating results per patient and per site. For example, the mean value of all systolic blood pressure measurements of a patient (per case) and an overall average value for the entire hospital (per site). The openEHR specifications enable us to generate advanced MMs like checking for contradicting entries stating presence and exclusion of a certain diagnosis at the same time. openCQA can generate such MMs semi-automatically. We did not include such MMs to avoid bias due to leaking prior knowledge about the DQ-issues (ET knew that there is one issue involving contradicting entries). Applied MMs included:

- Count of compositions for each case and the overall count of compositions per site.
- Currency of all compositions, i.e. the timespan between documentation time and current date/time for each patient and per site.
- The number of values for each variable, per patient and per site.
- Minimum, maximum, median, mean, standard deviation, lower quartile and upper quartile for numeric variables (overall per site and per case)
- Minimum-, maximum-, median- and mean-density (distance between timepoints) for timestamps (overall and per case)

All MMs applied during DQA were stored as a reproducible knowledge base ([27], commit c0a8a784, file kb_ML_Knowledge.json). Such a knowledge base unambiguously defines an applicable compilation of MMs. The script in Additional file 2: Appendix B automated calculations for MM-results and their export. To simulate a collaborative DQA-knowledge learning process, MM-results were calculated and exported as if each fictive site had done this independently (using the same knowledge base). openCQA was also used to aggregate the outcome values per case and per site (used MMs for outcome aggregation are part of the commit in file kb_Outcome_Measures.json).

#### Inspecting MM-results using the GUI

**Fig. 2:**
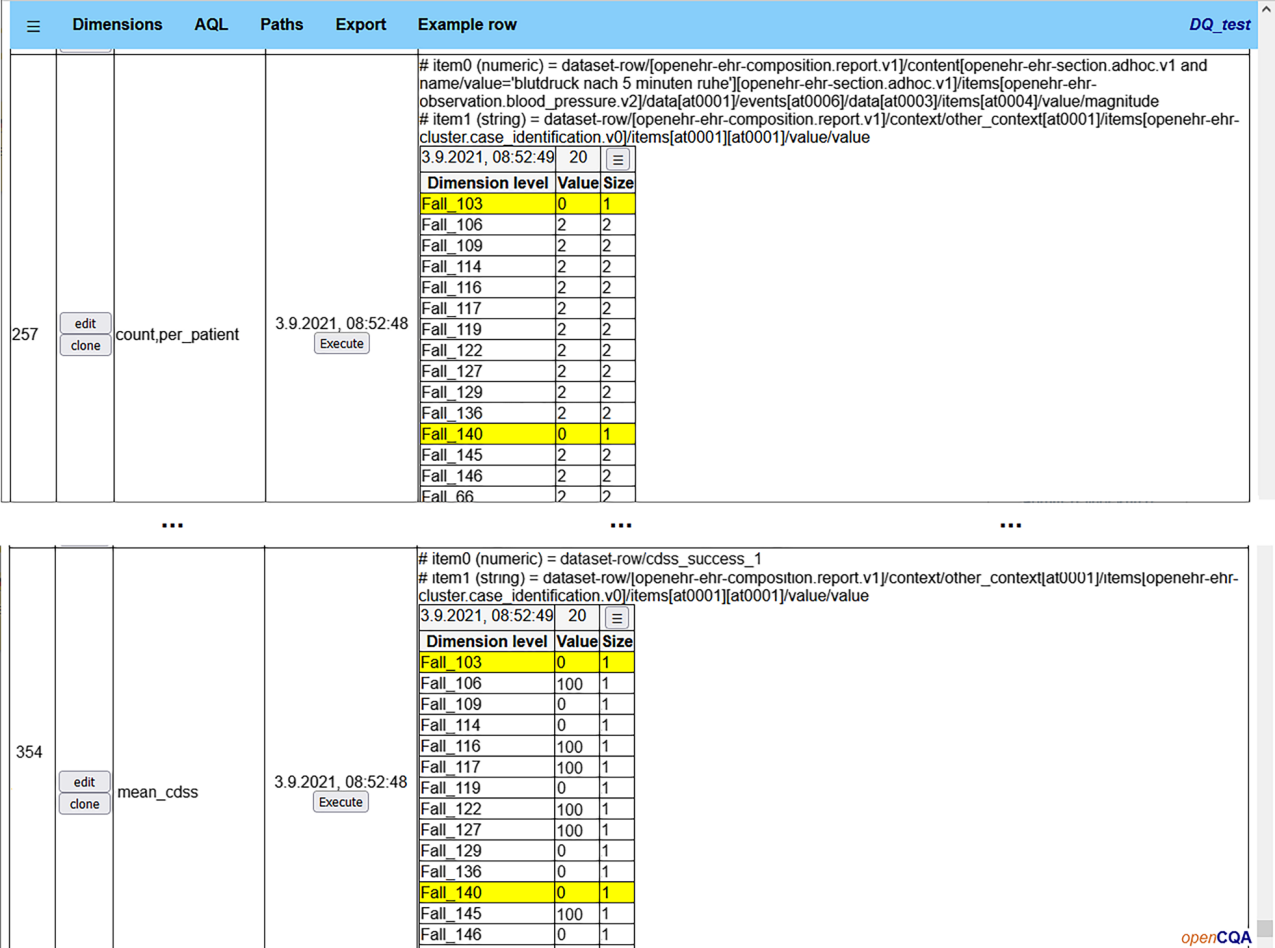
Example GUI-view on MM-results tables. Yellow rows mark cases without blood pressure values

Next step was to inspect MM-results to check if built-in DQ-issues and expected dataset characteristics were observable. These would be unknown in a real world use case. By doing this, we also verified correct processing of the data and ensured that the MM-results contained the required information that gives the machine learning algorithm the possibility to identify the DQA-knowledge. ET inspected the MM-results using the GUI of openCQA. While checking if a built-in DQ-issue was observable, ET also rated the difficulty of identifying the DQ-issue’s influence on the CDSS success when using the GUI without subsequent machine learning. This subjective rating based on two questions: 1. Is the issue easily identifiable as DQ-issue in the displayed MM-results? For example, a count of zero for a mandatory variable’s value is an obvious problem, whereas an 8 days old composition is not suspicious. 2. Is it difficult to identify the relationship between the issue and the effect on outcome using the GUI? Figure 2 illustrates how MM-results were displayed. An issue that causes the fictive CDSS to fail produces results tables in which each line holding a problematic value in one table matches one line with CDSS success value zero in another table. It is reasonable to assume that a human can identify such a relationship. In contrast, if the issue just causes a slightly lower performance, only a targeted statistical test between the results in both tables uncovers this effect.

#### MM-results export

We used the export functionality of openCQA to export the MM-results (MM-results contained both aggregated outcome data and MM-results about the clinical data). We concatenated the exports from all sites into one file for the following machine learning.

In the example of Table 1 the second to fourth columns hold the mean value of the correct/incorrect predictions, e.g. 53 if 53% of predictions were correct. We used shorter meaningful column names in Table 1. Names in the actual export were more verbose, e.g. “mean_ON_heigth_-1103246106_magnitude_1579900770” (numbers solely ensure unique names).

**Table 1:** Structure of concatenated MM-results exports for machine learning

Dimension	Outcome 1: mean CDSS success rate	…	Outcome 3: mean CDSS success rate	Composition count	…	Mean body height	Currency min composition	Heart rate value count
Overall	53	…	52	50	…	174.6	89,252	495
Per case	100	…	0	1	…	179	1,383,032	10
…	…	…	…	…	…	…	…	…
Per case	0	…	100	1	…	189	1,203,752	10
Overall	67	…	68	750	…	183.5	2836	982
Per case	100	…	100	1	…	190	1,724,492	8
…	…	…	…	…	…	…	…	…

The first column’s value indicates the dimension in which MM-results in this row were aggregated, i.e. for the entire site (overall) or for one patient (per case). Following columns hold aggregated MM-results. Each row with dimension “overall” marks the start of a new site’s export

### Data driven learning from MM-results

#### Machine learning

We chose decision trees (DTs) as machine learning method to explore the feasibility of data driven DQA-knowledge learning. DTs have the advantage to be a well-known method, they are easy to interpret and the implementation of MMs based on DQA-knowledge derived from DTs’ splits is straightforward. Furthermore, DTs have no special requirements for their input data, e.g. normalizing or centering of the data. We used rpart [28] as implementation of DTs in the language R. Analysis of variance (ANOVA) determined the best splits. The MM-results constituted the input variables for the decision tree (cf. Table 1 starting with column *Composition count* to the right). For each decision tree, we used one of the three outcome columns as label/training target (cf. column *Outcome 1/2/3: mean CDSS success rate* in Table 1). We used the entire dataset’s rows to train the model since the purpose of the resulting DT was its interpretation without evaluating the machine learning model’s performance.

The applied machine learning workflow consists of two phases. The first phase trains a DT on the data for the entire site to determine MM-results, i.e. columns of our training data, which provide relevant information aggregated per site. An example would be the number of dataset rows, i.e. the number of compositions in the data for one site. These variables are attached to the “per case”-data, e.g. in each row with results aggregated for a single patient a variable holding the number of compositions in the whole site is added. The second phase trains the DT on the data with MM-results aggregated per case (including the added relevant MM-results per site). Additional file 3: Appendix C provides the R-script of our machine learning workflow.

#### Deriving DQA-knowledge

**Fig. 3:**
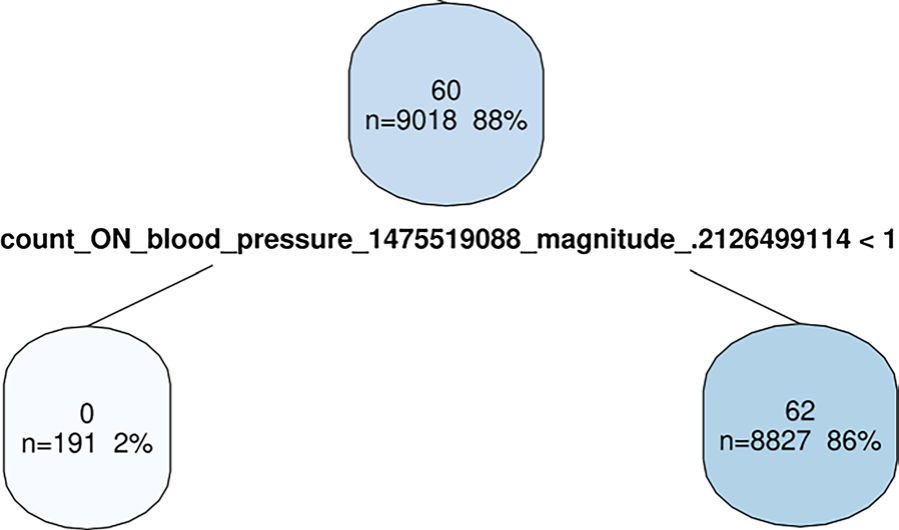
Example split from decision tree indicating the effect of missing blood pressure values. Node captions contain CDSS prediction accuracy in subset (e.g. 60), absolute (e.g. n = 9018) and relative (e.g. 88%) number of instances in subset. Splitting condition below node, left side fulfills the condition

Last step to learn DQA-knowledge from the exported MM-results was to interpret the tree, i.e. to derive rules covering the DQ-issues that influence the CDSS predictions. Figure 3 shows an example split from a decision tree. The split indicates that before the split was applied, the CDSS predictions for this subset of 9018 cases (88% of all cases) had 60% correct predictions. This subset was divided into two groups depending on the count of systolic blood pressure values per case, i.e. cases with no values (191 cases) and cases with one and more values (8827 cases). The subset of cases without blood pressure values had no correct predictions while the other subset had 62% correct predictions.

Thus, the split in Fig. 3 gives a hint to a DQ-issue (patient without blood pressure values) and its effect (CDSS fails). A knowledge base (applicable compilation of MMs for a certain task, cf. [11]) considering this derived DQA-knowledge could for example include an MM that lists all patients without blood pressure values (Additional file 4: Appendix D shows an example of such a MM for openCQA).

#### Comparing data driven learning results

One author (ET) applied the machine learning workflow three times, each time using another of the generated outcomes. This author knew the DQ-issues and their effect on CDSS predictions (cf. Fig. 1), thus, he was able to compare rules indicated by the DTs’ splits to the actual defined DQ-issues. This produced three ratings for each DQ-issue addressing the question, which DQ-issues the DTs covered. The other two authors (AW and NG) each conducted the DQA learning process including the DT interpretation once with *Outcome 1*, leading to a list of free text rules describing the derived knowledge about DQ-issues for each author. They both had no prior knowledge about the actual DQ-issues that were present in the data (not even the number or category of issues). We did this to accommodate for the first authors bias in interpreting the trees due to his prior knowledge about the issues. Finally, ET and each author (i.e. ET + AW and ET + NG) compared the free text rules to the gold standard list of DQ-issues present in the dataset. Later we refer to the result of this comparison as *Control*.

## Results

The generated dummy data, outcome data and MM-results are available ([29], folder “dummy data, outcome data, MM-results”). The numbers of counted DQ-issues affecting outcome generation are available in folder “triggered issue rules”. Counts were in the expected ranges. The exported MM-results used for machine learning can be found in file “MM-results_export_for_machine_learning.csv”. MM-results for 122 MMs were exported as 10,315 rows of data available for machine learning (20 for dimension per site, 10,276 per case and 19 rows had data on cases with missing case-id). Additional file 5: Appendix E contains the DTs resulting from machine learning on Outcomes 1 to Outcome 3.

Table 2 column *GUI* shows the results of manually inspecting MM-results in openCQA. The values for each DQ-issue indicate how well MM-results shown in the tool’s GUI indicate the respective issue and its effect. Columns *Outcome 1* to *Outcome 3* show the results from comparing the DQ-issues indicated by the DTs’ splits to the actual DQ-issues (knowing the truth). Values indicate how well the splits cover the respective issue and its effect. Column *Control* shows the results from learning DQA-knowledge without prior knowledge about DQ-issues. Value “no” indicates the DQ-issue was not derived. “yes” indicates both controls (AW and NG) derived this DQ-issue as influencing factor for the CDSS. “yes/no” indicates that only one of them derived the knowledge about this issue. The free text lists AW and NG created contained no rules where any of the authors had doubt or divergent opinions on how to rate it compared to the gold standard.

**Table 2:**
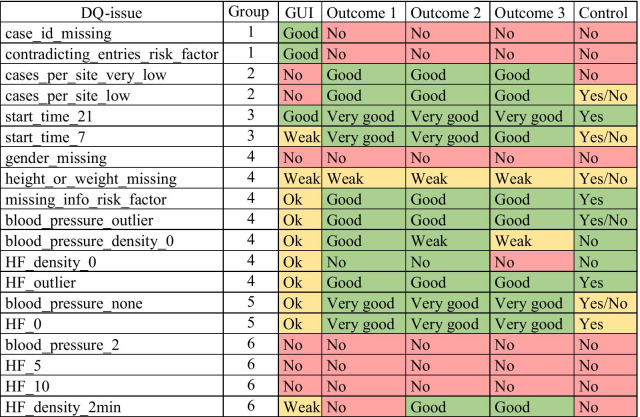
Overview DQA-knowledge learning success

The DTs for Outcome 1 to Outcome 3 had only minor differences. Of the 19 DQ-issues present in the data, eleven/twelve were visible in the DTs (prior knowledge). DTs contained only few splits in lower parts of the tree that indicated rules without justification in an actual DQ-issue. Four of the issues were found by both controls and five issues by at least one control, indicating that at least nine of the issues could be derived without prior knowledge. One of the controls derived one unjustified rule.

We perceived six groups of DQ-issues in our results on learning success as indicated by column *Group* in Table 2. (1) The issues case_id_missing and contradicting_entries_risk_factor are both obvious problems for humans inspecting MM-results. Thus, targeted MMs for checking them can be applied. To estimate the effect on CDSS performance, a human could perform a targeted statistical analysis regarding the correlation between affected cases and their CDSS outcome. We did not apply any targeted MMs in our work to avoid creating a bias due to prior knowledge about issues. Our simple machine learning approach did not consider cases with missing case-id since these were aggregated as one row during export. To identify cases with contradicting values in risk factor entries, the risk factor’s name is necessary (e.g. “diabetes”). The exported MM-results consisted of numeric values only, i.e. the export was missing the necessary information to distinguish cases with contradicting entries. Thus, manual DQA-knowledge learning for these two issues had good ratings and the machine learning supported approach was rated with “no”. (2) The issues cases_per_site_low and cases_per_site_very_low were not visible in the GUI, since data was not pooled (GUI only showed results for one site). The DTs had splits in prominent positions indicating these issues with satisfying precision for thresholds (e.g. row_count < 375 where actual issue threshold is 400). (3) Issues start_time_21 and start_time_7 were visible in the GUI when MM-results were aggregated per day (only applied in GUI, not exported for machine learning). start_time_21 was visible through a clear cutoff in aggregated outcomes per day, because predictions on data older than 21 days failed completely. For start_time_7 the cutoff was less obvious because predictions were only less accurate but did not fail completely. If the DQ was already bad, e.g. because the site had a low number of cases, the effect was hardly noticeable in the GUI. The DTs had splits in prominent positions with good precision for thresholds (e.g. currency ≥ 21 and currency ≥ 7. For Outcome 3 the split for 7 day rule was shown as ≥ 8). (4) For gender_missing, height_or_weight_missing, missing_info_risk_factor, blood_pressure_outlier, HF_outlier, blood_pressure_density_0 and HF_density_0 the odd values are apparent for humans (e.g. missing values, a systolic blood pressure > 300 or a density of 0, where the density of 0 means that all timepoints for different measures of a vital parameter have the same timestamp). However, to identify the correlation, for each issue it would be necessary to compare two tables with 50–1000 rows, i.e. one table with the MM-results per case and the CDSS prediction accuracy per case (cf. Fig. 2). Thus, some effects may be overlooked. Dependent of the strength of the effect and other confounding issues, a statistical test is likely to show a significant difference (e.g. height_or_weight_missing) or not (e.g. gender_missing). The DTs contained splits for the DQ-issues which had stronger effect in the respective subset of the data (e.g. heigth count = 0, missing_info_risk_factor, blood pressure magnitude ≥ 274, blood pressure density < 334, heart rate standard deviation or maximum value). Some DQ-issues with weak effect were not visible in the DTs but were listed in alternative splits, indicating that the impact of the issue was too low to be selected as split but maybe some advanced machine learning methods would be able to detect them (e.g. gender_missing, HF_density_0). For the height_or_weight_missing rule, only a split on height is present in the DTs because weight was missing less often. (5) For blood_pressure_none and HF_0, a human would need to compare two big tables, but since the effect is very strong (CDSS fails), we deemed it as noticeable. The DTs showed a split in prominent positions in the tree (blood pressure count or heart rate count < 1). (6) The last group of issues (blood_pressure_2, HF_5, HF_10, HF_density_2min) did not stand out with obviously odd values. Furthermore, their effect was weak. Thus, statistical tests may not be significant if correlations with outcome were investigated. Since the effects were small, the DTs only contain splits indicating HF_density_2min in two of three DTs. blood_pressure_2 and HF_5 were visible in alternative splits but not shown in DTs.

## Discussion

Finding suitable MMs for DQA adjusted to a certain task and defining reference ranges that indicate noteworthy MM-results is typically resource intensive and often based on personal experience of involved experts. Our research explored the applicability of a data driven approach with the aim to support learning of tangible DQA-knowledge. Our results show that even simple machine learning methods can derive sensible knowledge from exported MM-results and respective outcome data. The derived rules sensibly complemented the rules likely to be found in a manual approach.

### Sensible DQA-knowledge

We applied DTs to support deriving knowledge about DQ-issues and their effects. DTs indicated DQ-issues with satisfying values for their respective thresholds. Researchers without prior knowledge about the DQ-issues (control) were able to derive sensible rules. DTs contained only a few nonsense splits in lower parts of the trees. Control derived only one false DQ-issue rule. When control shared their individually found rules, both noted a lack of context about the original data and the interpretation of the exported MM-results. This was due to the efforts to prevent knowledge leaking about DQ-issues, i.e. keeping control out of data generation and manual DQA (cf. Fig. 1). A lack of context could handicap the identification of rules from the DTs, but there is no reason to assume that it could help. Thus, a bias from this would distort our results towards less success, i.e. it does not impair our finding, that the presented approach is suitable to identify sensible DQA-knowledge.

The data, DQ-issues and how issues influenced the outcome were fictive. Furthermore, we pragmatically created rules defining how DQ-issues affect the outcome because these were straightforward to check in outcome generation and to compare with DQA-knowledge learning results. Real world data is complex, it may not be possible to model a DQ-issue’s effect (e.g. as a rule) and probably many confounding factors regarding outcome exist. To address this, we performed the probabilistic outcome data generation with probabilities based on DQ. Thus, DQ-issues had no pure effect on the outcome. Central idea is that in some cases there may be a correlation between some MM’s result value (indicating an issue) and the outcome. Motivation of our work is to provide an approach that helps to identify such relations between MMs’ results and outcomes. Testing our method in an artificial setting was essential, since experience shows “[…] it is too easy to ‘get a result’ in the data science space. […] and even more likely that one can then come up with some ‘intuitions’ to rationalize the results.” [30]. Thus, we needed a truth to check our learned DQA-knowledge. This check, i.e. comparing our learned DQA-knowledge to the actual defined DQ-issues to determine values for Table 2, involved a subjective rating (e.g. rating the split displayed in Fig. 3 as “very good” indicator for the DQ-issue “blood pressure none” and its effect of a failing CDSS). Therefore, we justified the values in the results section. Even with this small subjective factor, we deem the check as robust enough to substantiate the finding that the derived rules were accurate. In a real world use case, no instance verifying the correctness of the learned DQA-knowledge exists. Our results with artificial data give us some confidence that our approach indicates sensible issues and thresholds and only few misleading results. We are aware that this is no guarantee for sensible results in real world use cases, but we think this is not critical, since we see the value of our approach in indicating interesting measures and thresholds to consider and not in taking any automated decisions. The consequence if our approach fails in a use case is that some irrelevant measures for DQA are proposed.

DQ-tools like openCQA provide us with functionalities to quickly generate many MMs, e.g. by generating MMs based on datatype, based on constraints from information models, by adding aggregations in sensible dimensions, by adding MMs resulting in plots, relative values and more. Our approach cannot relieve the expert completely from the burden to select sensible MMs for DQ-assessment. But it can help the expert by allowing to automatically generate common statistical measures and constraint checks aggregated in sensible dimensions and then assisting in the identification of the relevant MMs and result thresholds for certain tasks. Our approach can be applied to all MMs with results that can be transformed into a structure like Table 1. By cooking down the results of 122 MMs to 12 relevant MM-results our DQA-knowledge learning approach already provided a valuable prioritization. Respective thresholds for the MM-results additionally enable to support DQA by highlighting only the MM-results with relevant values. Beyond that, DTs are hierarchical structures. They build the tree structure by repeatedly seeking for binary splits explaining the most variance in the current data subset. Thus, splits in the upper part of the tree indicate the DQ-issues causing the most effect on the outcome. That way DTs provide an additional prioritization of MMs. We are aware of only one other work that considers the learning of task dependent prioritization for MM-results [17]. While Johnson et al. aim to quantify the effects of changes in DQ, they proposed a method that calculates weights for MMs on certain variables. This could also serve as prioritization. Because these weights base on a linear regression, their method only works for MMs with results that suit the requirements for linear regressions. Many intuitive conceptions of DQ do not define good or bad DQ in a linear way where a low MM-result value is bad and a higher value is steadily indicating better DQ. For example, the number of heart rate measurements per case should be in a normal range. Having no values would be bad for our fictive CDSS, having more than ten would be good, but having 1000 values for a single case would be odd again (even the range from one to ten measures per case was not linear in our scenario). Our approach allows to identify reference ranges that indicate MM-results that need attention. Thus, it is an important addition to currently published methods.

### Complementing DQA-knowledge

The results indicate that our applied learning approach is suitable to complement DQA-knowledge with new insights (compared to manually derived knowledge during DQA). From comparing the results in Table 2 column *GUI* (manual approach) to the results based on machine learning (columns *Outcome 1* to *Control)*, we identified three groups of issues where adding our approach was beneficial. (1) Combining the MM-results of multiple sites enabled us to identify DQ-issues that were not visible in results from just one site (low and very low number of cases per site). Sharing MM-results instead of patient data can be an advantage for collaborative learning of DQA-knowledge, although sharing of MM-results as well needs attention to ensure that no privacy issues occur. (2) If values were obviously odd (e.g. missing value or outlier) or had a strong effect on the outcome (e.g. CDSS fails without blood pressure values) a human inspecting MM-results could notice these issues, but we noticed a risk of overlooking rare effects in big result tables. Furthermore, to investigate the effect of the DQ-issue on the outcome in most cases a statistical test would be necessary. Thus, the manual approach is more labor intensive and error-prone for such issues. (3) We deemed DQ-issues without obviously odd values and without obvious effect on outcome (e.g. compositions that were older than 7 days) to be likely to be overlooked in the manual approach. To identify a correlation between such a DQ-issue and its effect on the outcome, a human needed an anticipation to trigger a targeted statistical test investigating the correlation. DTs basically perform automated statistical tests (ANOVA) on each variable and possible splitting value. By this, the need for anticipating DQ-issues is removed improving the identification of such issues.

In our results, the DQ-issues case_id_missing and contradicting_entries_risk_factor were found to be obvious for humans but not detected using DTs. Another example where other approaches are more suitable would be typing errors. In strings with a limited amount of eligible values, these are easy to identify manually by listing all unique values and the number of their occurrences. Data driven methods that work directly on the data are also suitable to identify cases (e.g. [18], 19) or distributions (e.g. [20]) that somehow deviate from “normal”. However, these approaches do not consider the task. For example, a composition that is older than 21 days or a patient with less than x measurement values is nothing that deviates from normal, but it is a relevant DQ-issue if we consider the task. As we can combine DQA-knowledge that we derive with our approach with knowledge from other approaches, manual or data driven, combining approaches to complement each other seems favorable.

## Conclusions

Our machine learning supported approach for task dependent learning of DQA-knowledge is suitable to derive sensible and practical knowledge about relevant MMs and their reference ranges for interpreting MM-results. The derived DQA-knowledge can sensibly complement other currently common approaches by closing blind spots for DQ-issues with undeniable practical relevance. For example, DQ-concepts that do not create MM-results suitable for linear regressions or task dependent DQ-issues that are not identifiable as obviously abnormal values. Our approach derives applicable knowledge for task dependent DQA, e.g. which measurable requirements does a local data stock have to fulfill to apply a given CDSS with good prediction accuracy? To the best of the authors’ knowledge, this is the first work on an approach providing tangible answers to such questions.

Although our approach provides task dependent DQA-knowledge it is flexible regarding the studied task, i.e. whether the task is a CDSS system or some other data use is not important. For initial learning of DQA-knowledge the task has to provide values about the task dependent DQ of subsets of the dataset. These values constitute the label data in the training instances for the machine learning model. DTs can deal with numeric as well as with textual labels (class labels). An obvious future research direction would be to apply our approach on textual DQ-issue labels. Other important future research directions concern the applicability of our approach in real world use cases and the elaboration of advanced machine learning methods for the learning process.

## Supplementary Information

**Additional file 1**. Overview DQ-issues.

**Additional file 2**. Data generation.

**Additional file 3**. Machine learning workflow.

**Additional file 4**. Example MM to check missing BP.

**Additional file 5**. Decision trees.

## Data Availability

The artificial data, outcome data and MM-results are available at https://doi.org/10.24355/dbbs.084-202107061200-0. Source code of applied tools is publicly available at https://gitlab.plri.de/tute/openehr-dummy-data-generator and https://gitlab.plri.de/tute/openehr-dq

## Abbreviations

CDSS: Clinical decision support system
DQ: Data quality
DQA: Data quality assessment
DT: Decision tree
MM: Measurement method
