# Supplementary figures and images for: A data driven learning approach for the assessment of data quality
Source: BMC Med Inform Decis Mak. 2021 Nov 1;21:302. doi: 10.1186/s12911-021-01656-x (PMC8561935; doi:10.1186/s12911-021-01656-x)

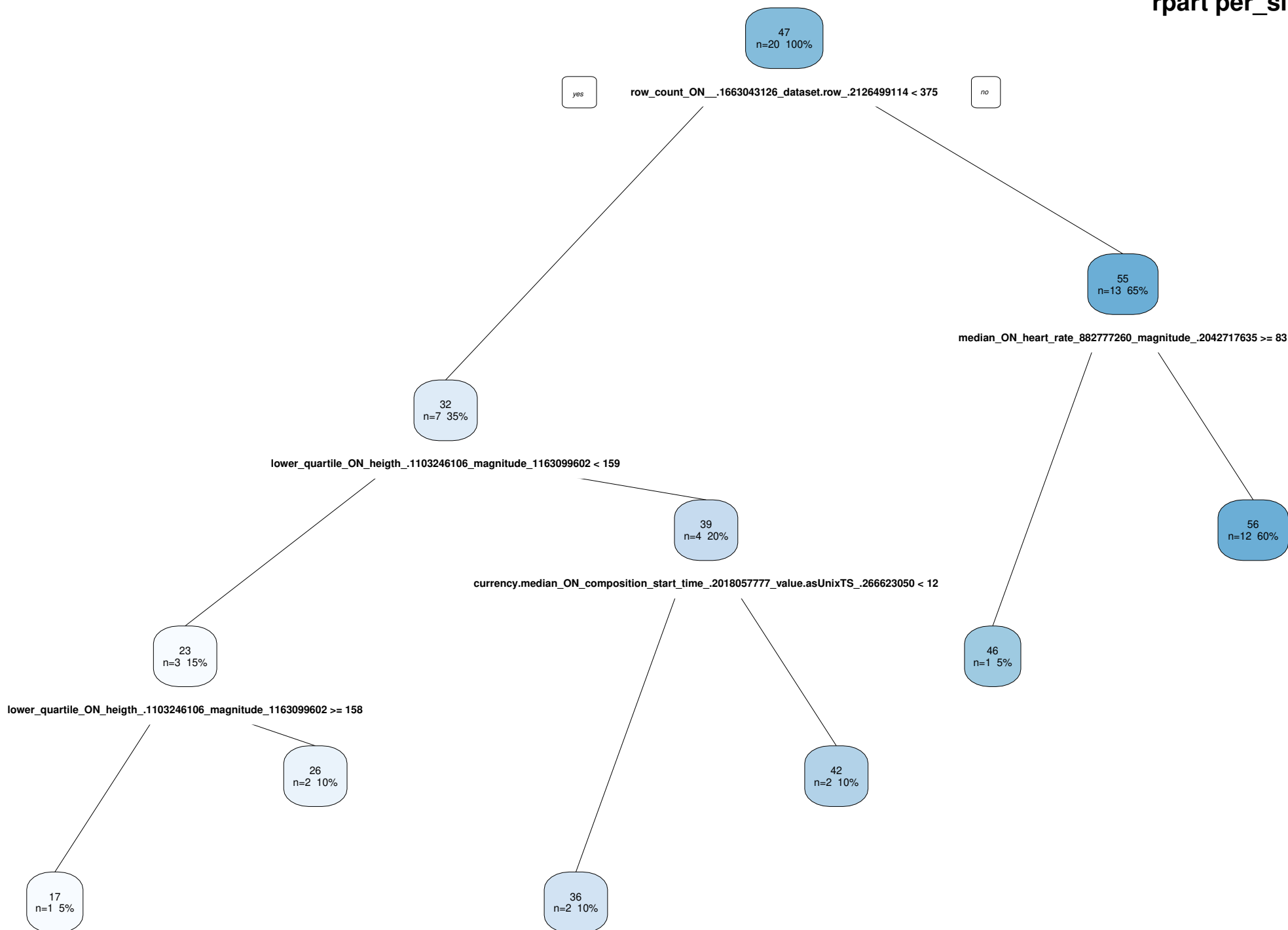



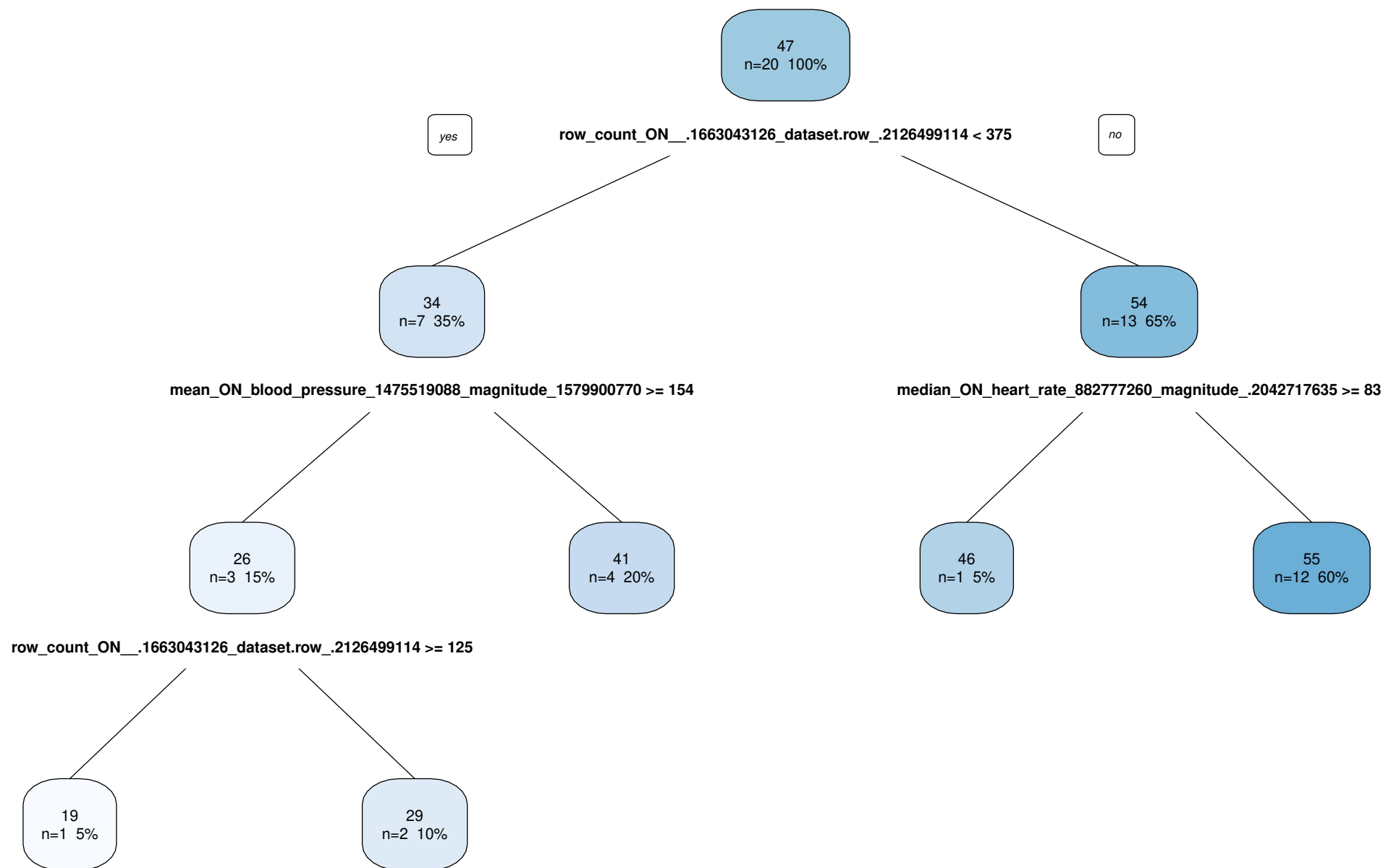

# rpart per\_case 2

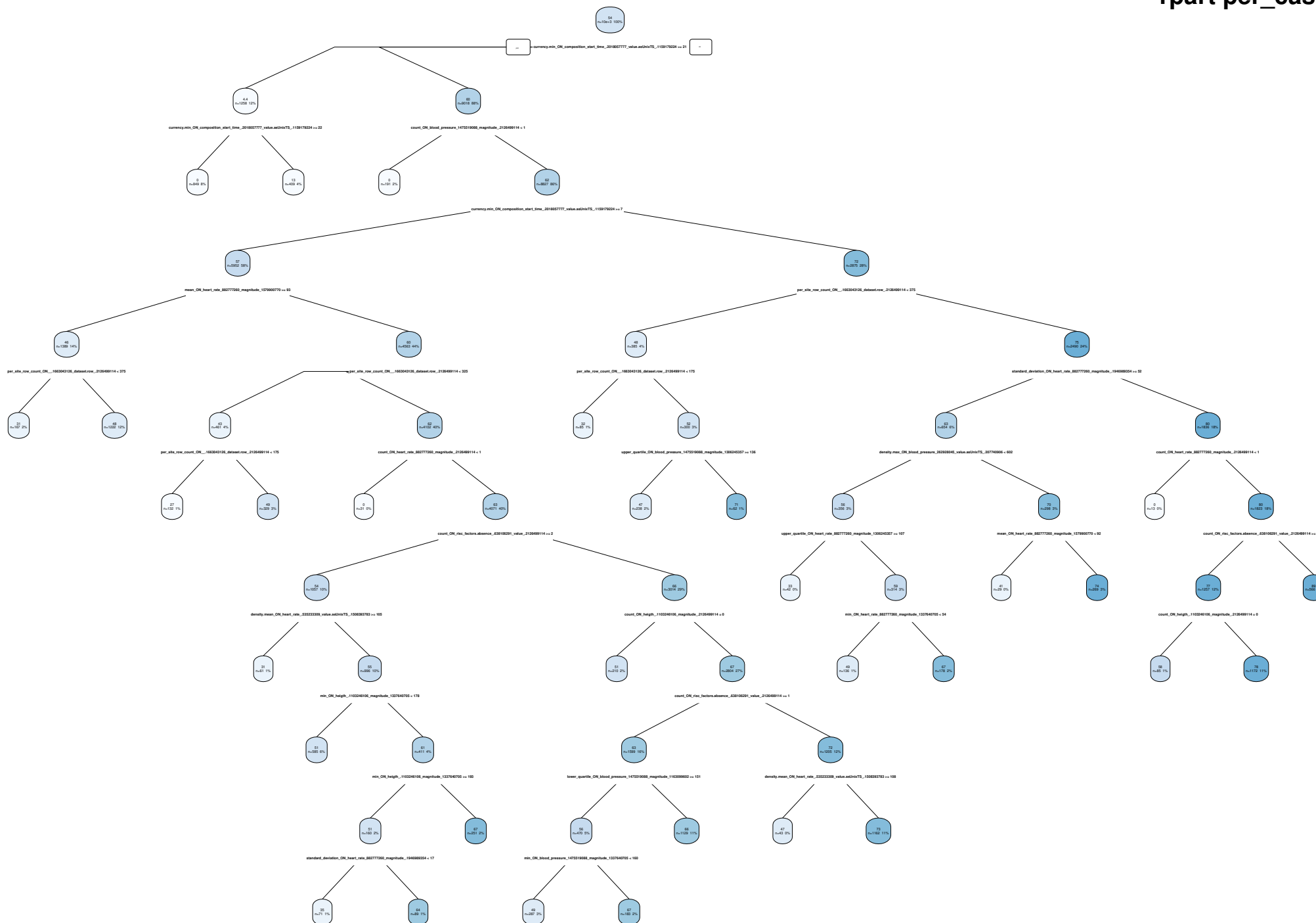

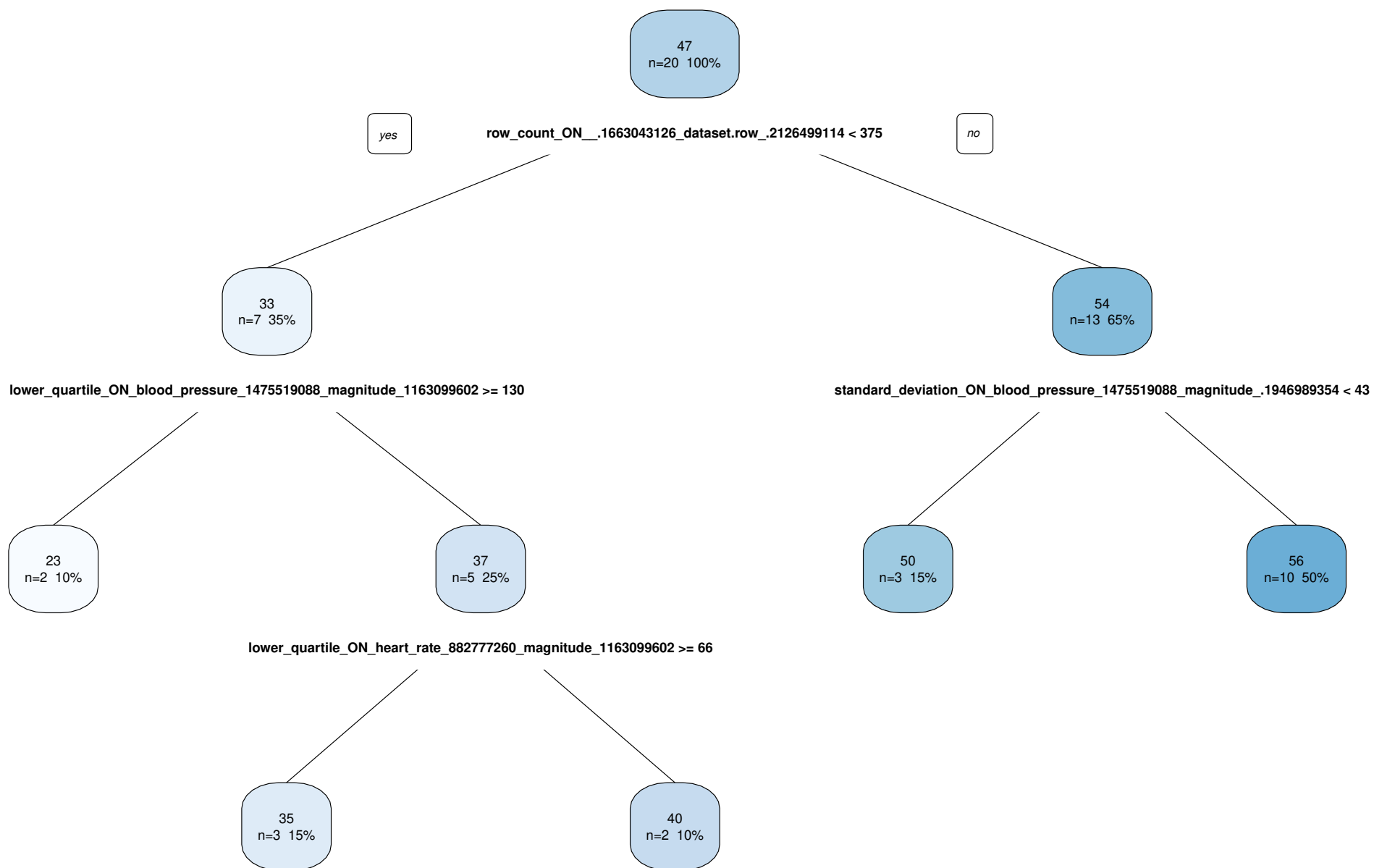

Supplement: Supplementary file 5 — Additional file 5. Decision trees. [file 12911_2021_1656_MOESM5_ESM.pdf]
